# Supplementary material for: Serum-Exosome-Derived miRNAs Serve as Promising Biomarkers for HCC Diagnosis
Source: Cancers (Basel). 2022 Dec 29;15(1):205. doi: 10.3390/cancers15010205 (PMC9818484; doi:10.3390/cancers15010205)
Supplement: Supplementary file 1 [file cancers-15-00205-s001.zip › Supplementary material S1.pdf]

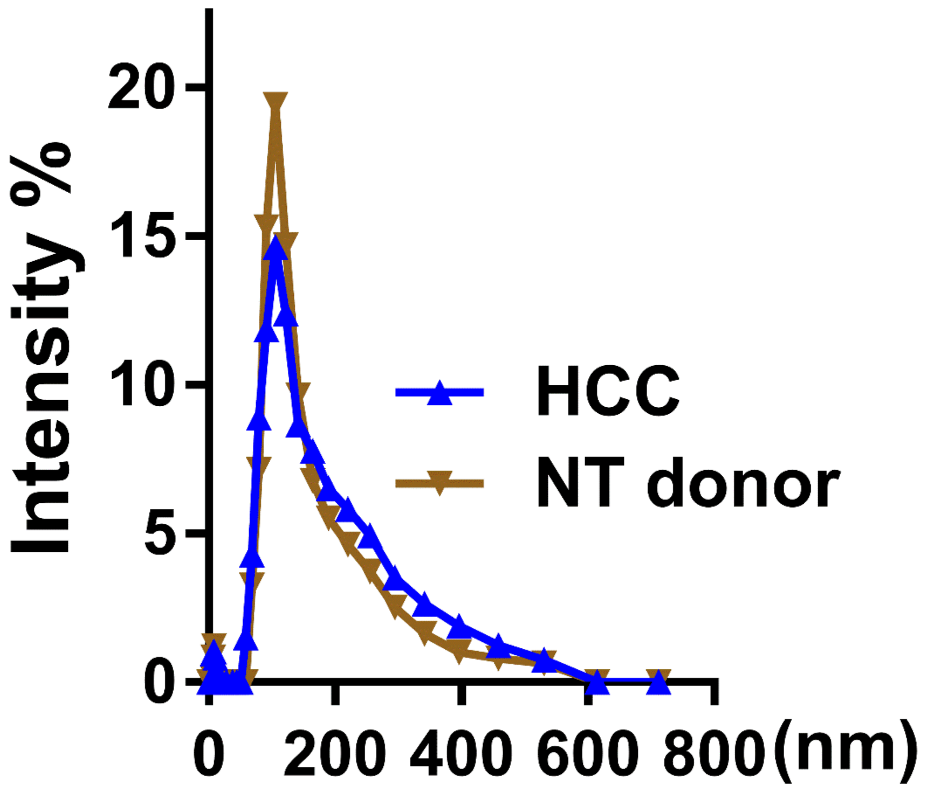

Figure S1: The hydrodynamic diameter of serum exosomes extracted from HCC patients and non-tumor donors.

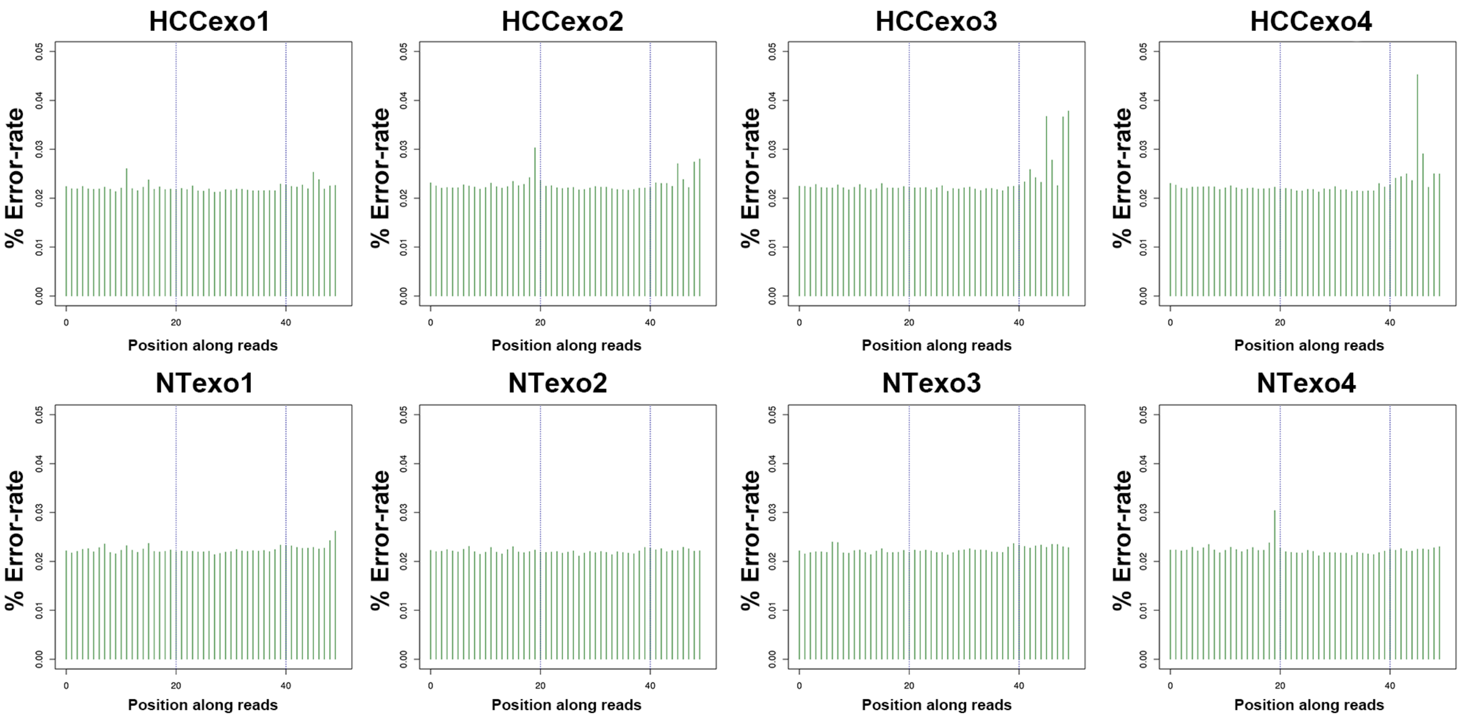

Figure S2: The error-rate of each base less than 0.05% representing the high quality of sRNAs sequencing.

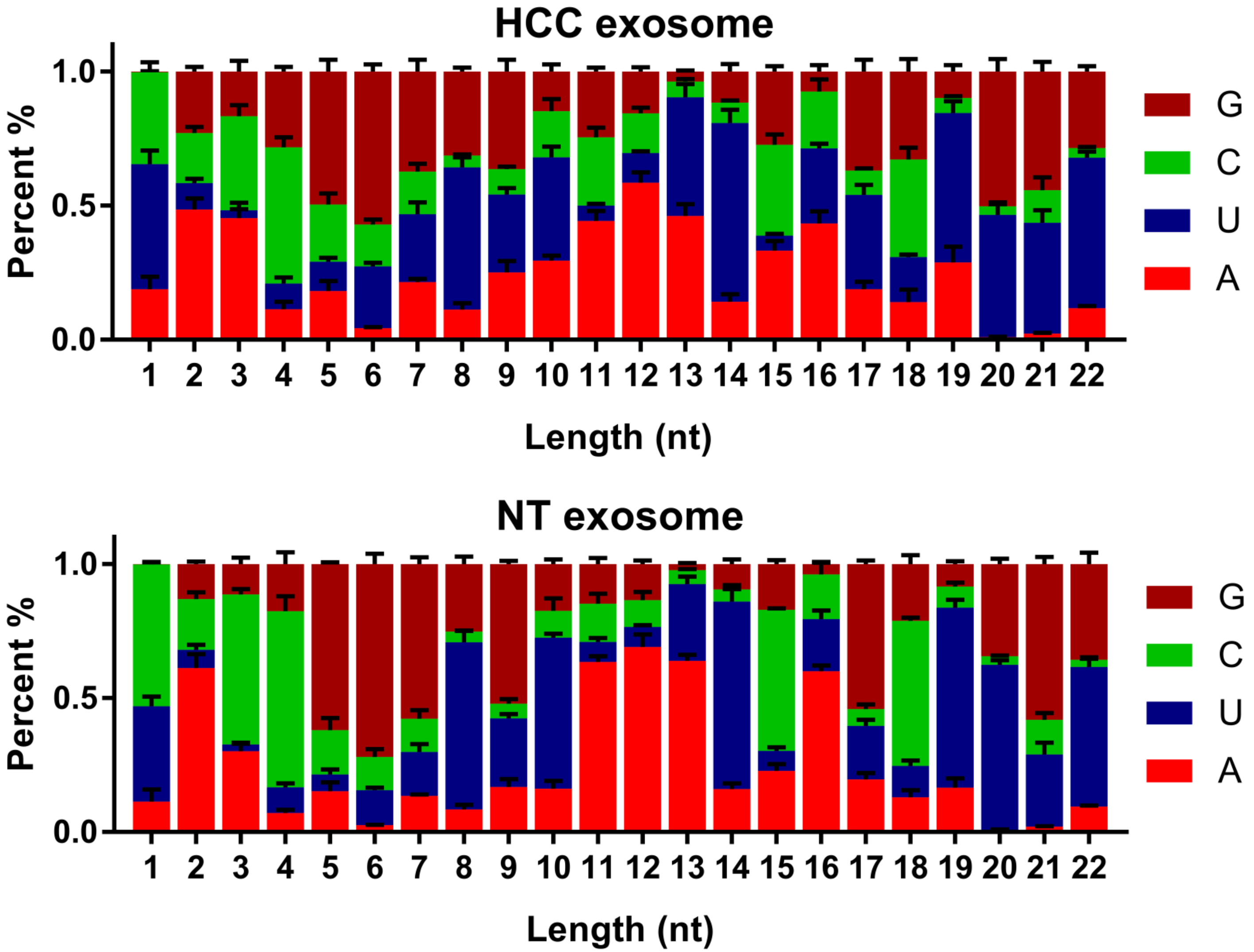

Figure S3: Base bias of serum exosome-derived miRNAs from 4 HCC patients and 4 non-tumor donors.

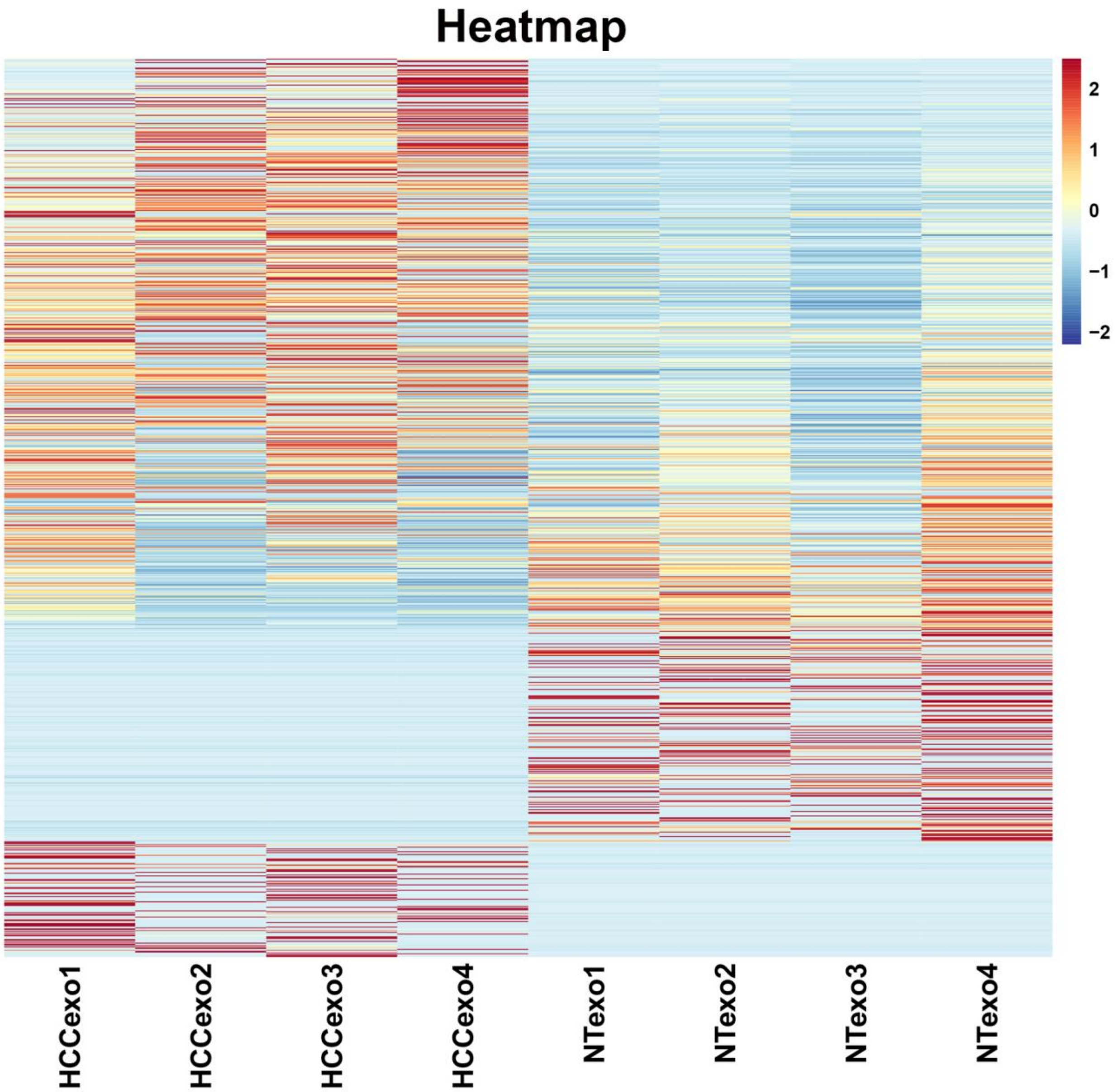

Figure S4: Heatmap depicts the expression of serum exosome-derived miRNAs in HCC patients and non-tumor donors.

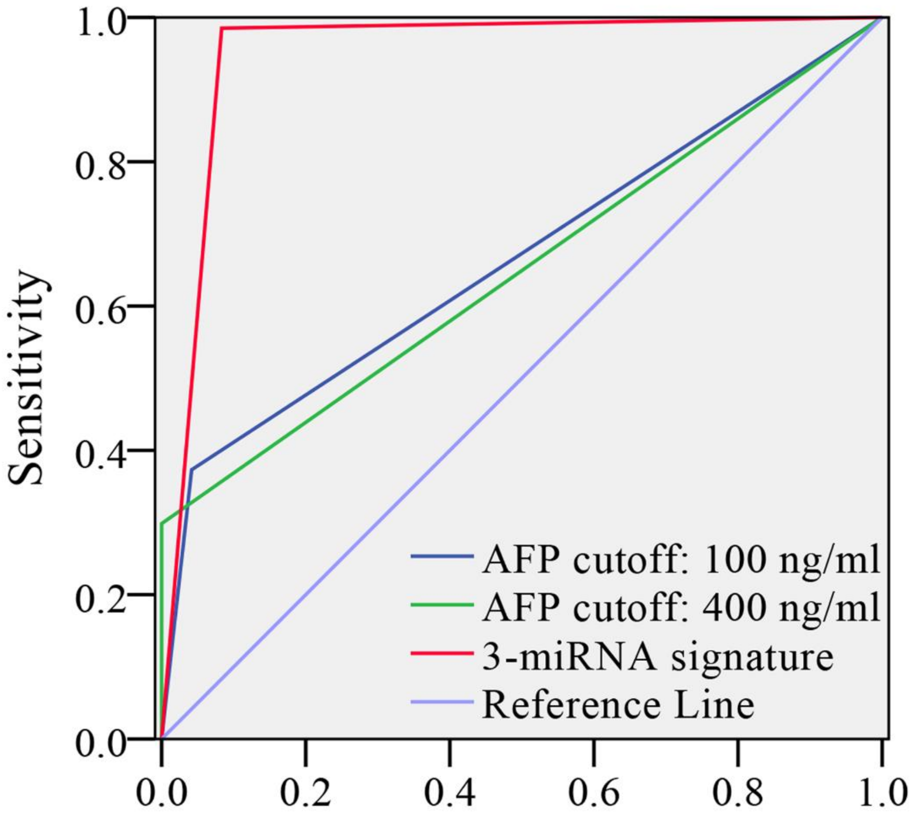

AUROC values for HCC diagnose

	AUROC	95% CI	P value
AFP cutoff: 100ng/ml	0.666	0.552 - 0.780	0.016
AFP cutoff: 400ng/ml	0.650	0.535 - 0.764	0.031
3-miRNA signature	0.954	0.892 - 1.000	< 0.0001

AUROC: areas under receptor-operated curve;

CI: confidence interval

Figure S5: The area under receptor-operated curve (AUROC) values of 3 serum exosome-derived miRNAs and AFP with cutoff of 100 ng/ml or 400 ng/ml on the diagnosis of HCC.

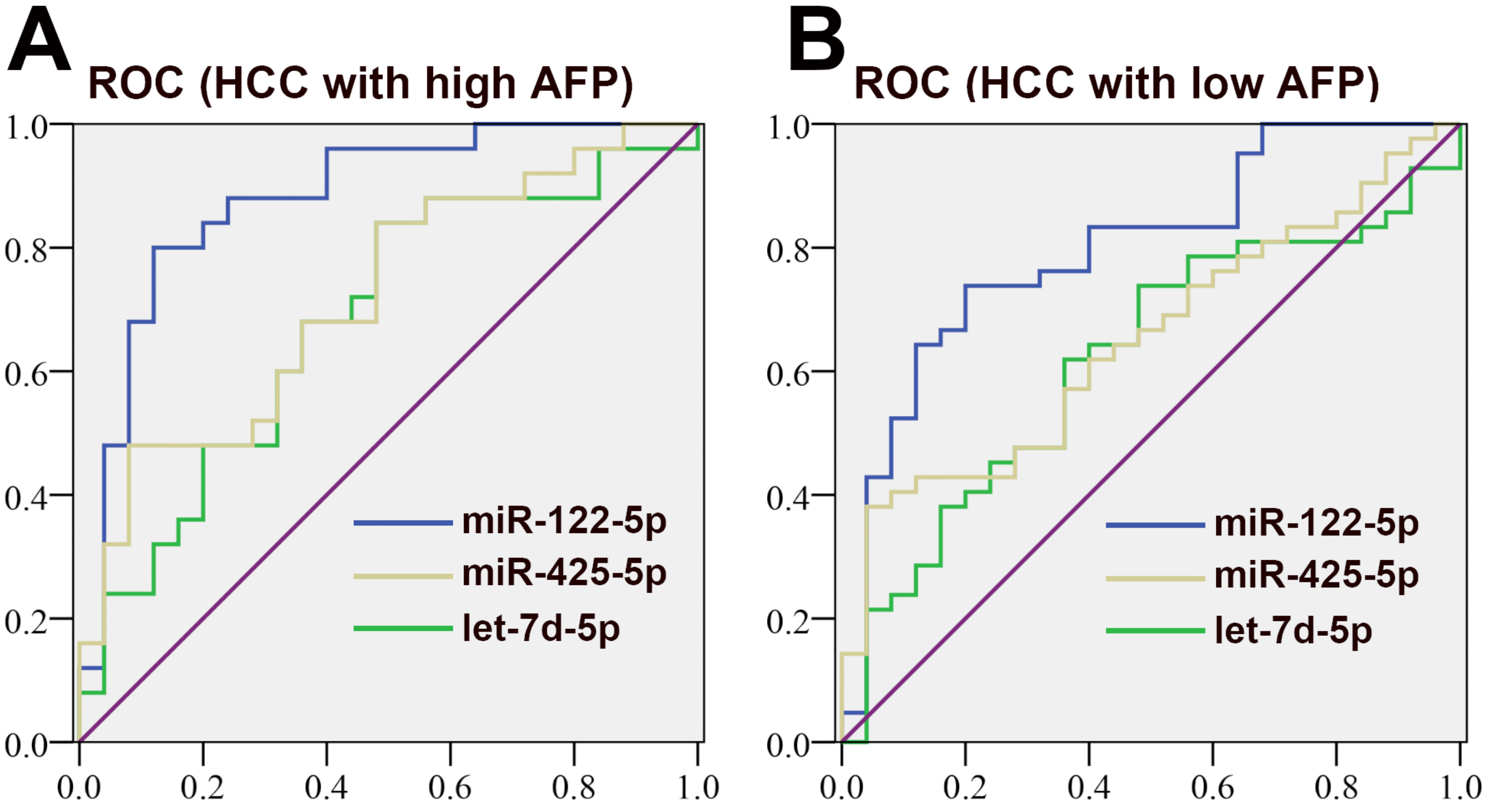

AUROC of 3 miRNAs for HCC diagnose (high AFP)

	AUROC	95% CI	P value
miR-122-5p	0.880	0.782 - 0.978	< 0.0001
miR-425-5p	0.682	0.548 - 0.831	0.028
let-7d-5p	0.720	0.579 - 0.861	0.008

AUROC of 3 miRNAs for HCC diagnose (low AFP)

	AUROC	95% CI	P value
miR-122-5p	0.800	0.690 - 0.910	< 0.0001
miR-425-5p	0.619	0.482 - 0.756	0.105
let-7d-5p	0.650	0.519 - 0.780	0.042

Figure S6: The area under receptor-operated curve (AUROC) values of 3 serum exosome-derived miRNAs on the diagnosis of HCC with high and low AFP.

Training set

HCC with low stage vs. non-tumor

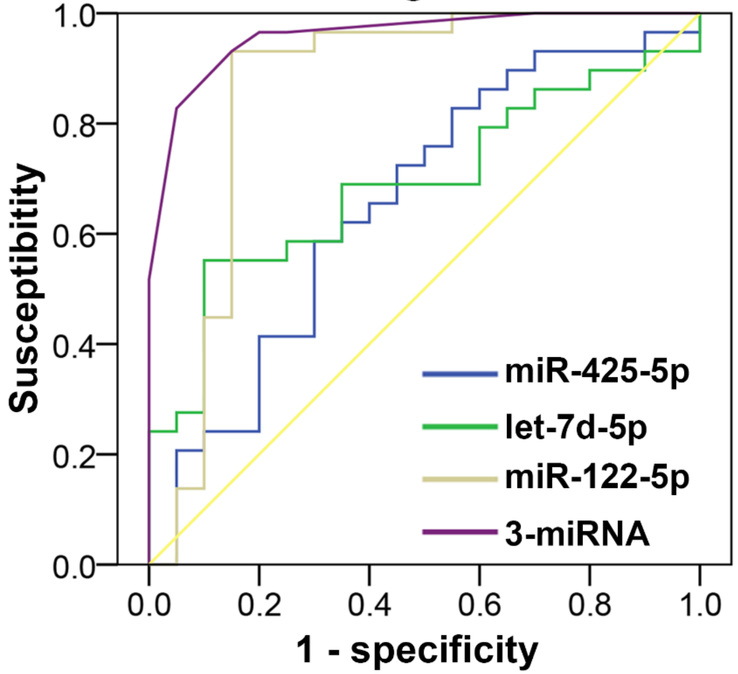

HCC with high stage vs. non-tumor

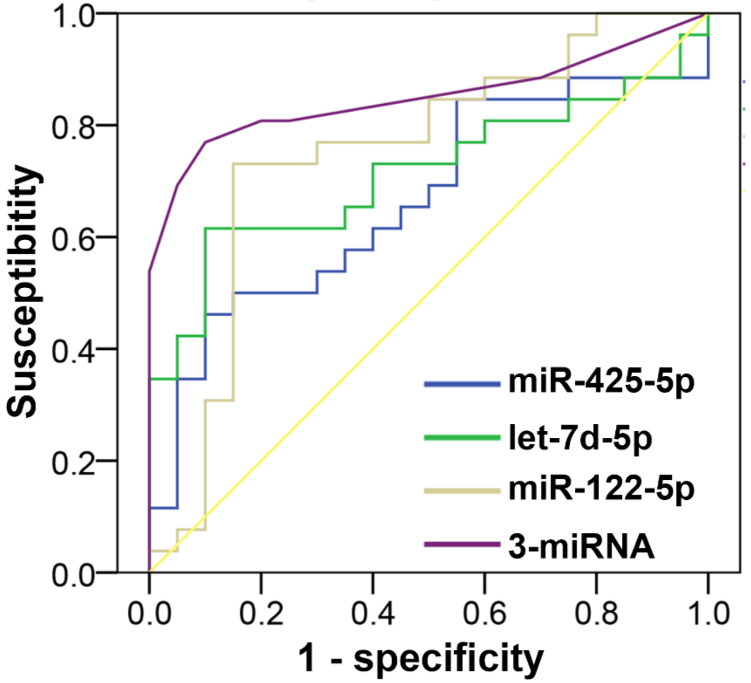

AUROC values in the HCC with low stage from training set

	AUROC	95% CI	P value
miR-122-5p	0.860	0.728 - 0.992	< 0.0001
miR-425-5p	0.655	0.495 - 0.815	0.067
let-7d-5p	0.690	0.541 - 0.838	0.025
3-miRNA signature	0.959	0.909 - 1.000	< 0.0001

AUROC values in the HCC with high stage from training set

	AUROC	95% CI	P value
miR-122-5p	0.750	0.598 - 0.902	0.004
miR-425-5p	0.665	0.507 - 0.824	0.057
let-7d-5p	0.715	0.565 - 0.866	0.013
3-miRNA signature	0.850	0.733 - 0.967	< 0.0001

Figure S7: The area under receptor-operated curve (AUROC) values of 3 serum exosome-derived miRNAs on the diagnosis of HCC with low and high stage (training set).

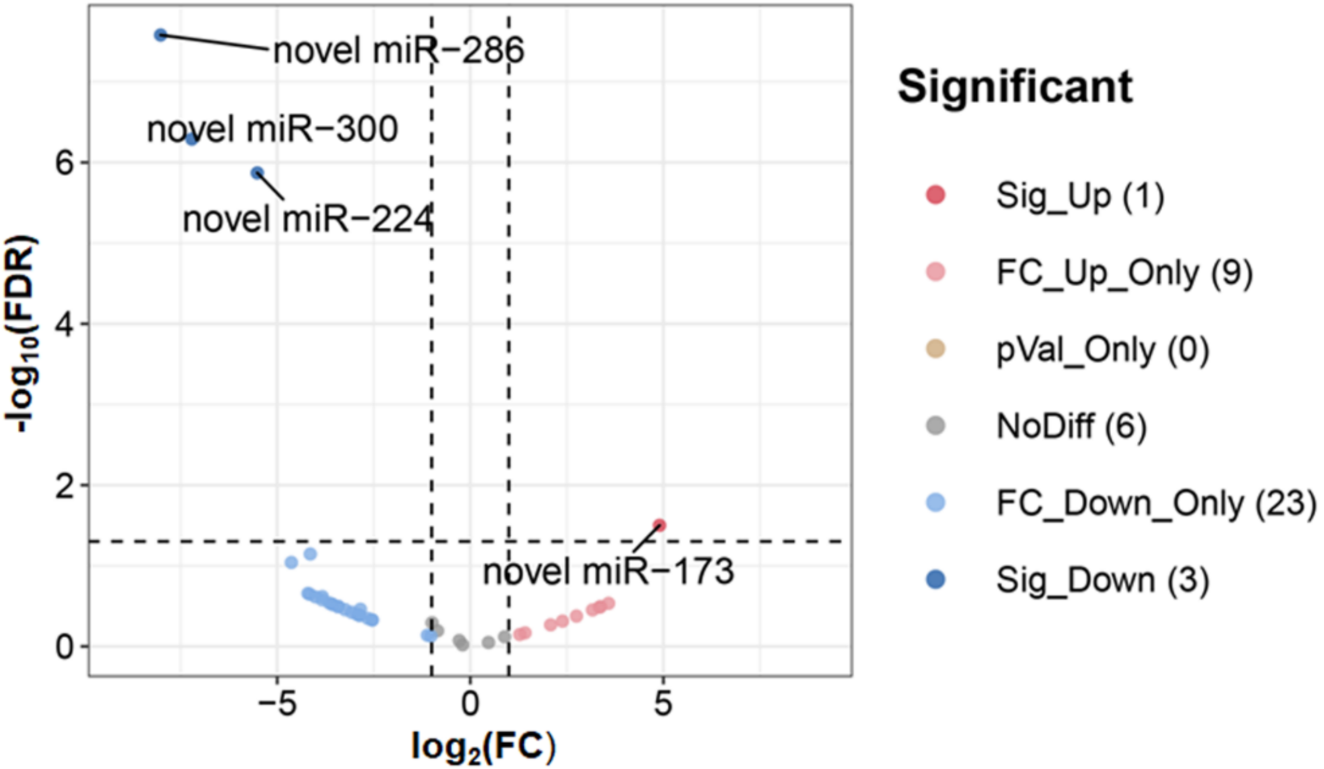

Figure S8: Volcano plot depicts differentially expressed novel miRNAs between the 4 HCC patients and 4 non-tumor donors

Novel-miR-286 **Novel-miR-300** **Novel-miR-224**

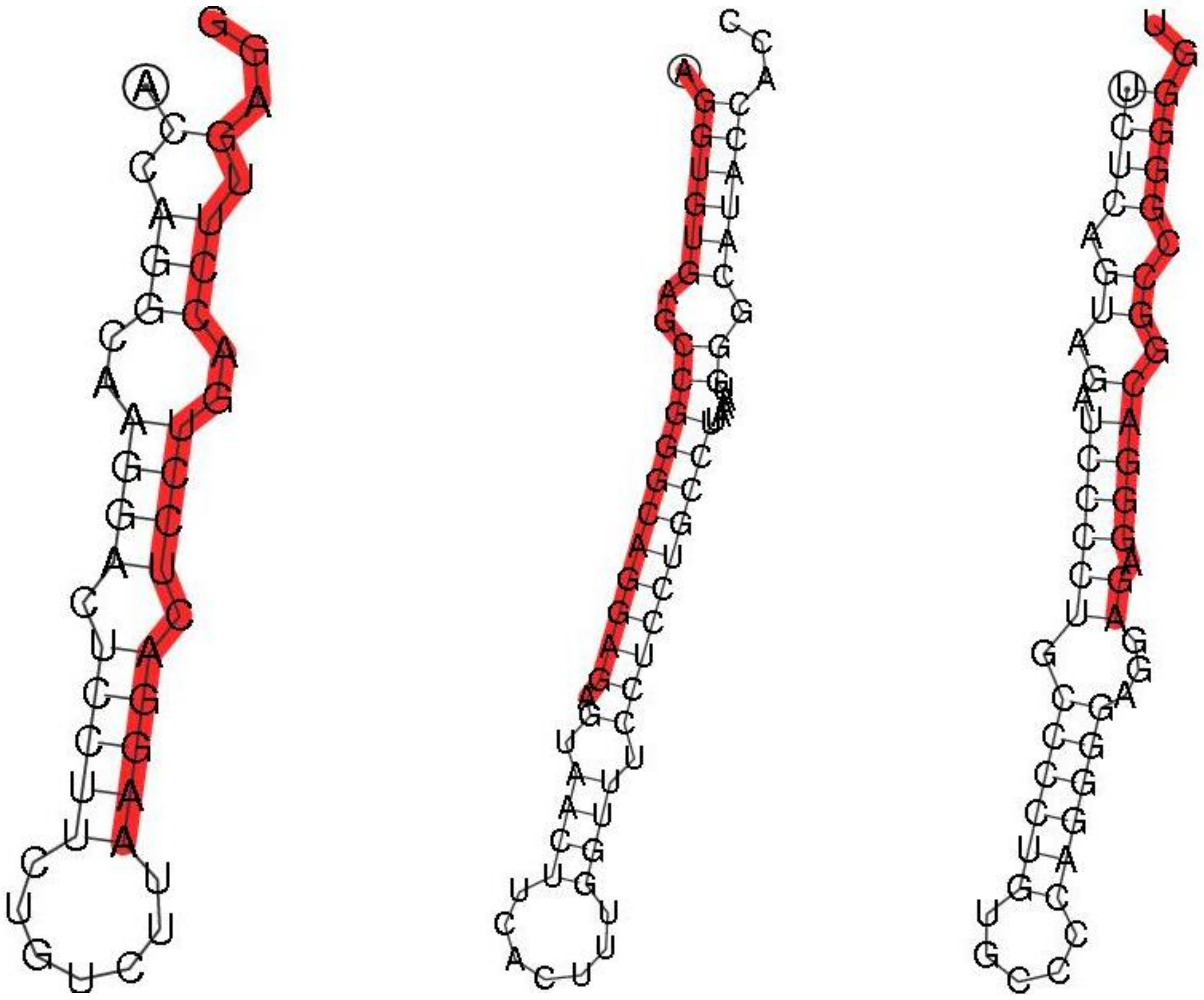

Figure S9: The structure of three differential expressed novel miRNAs.

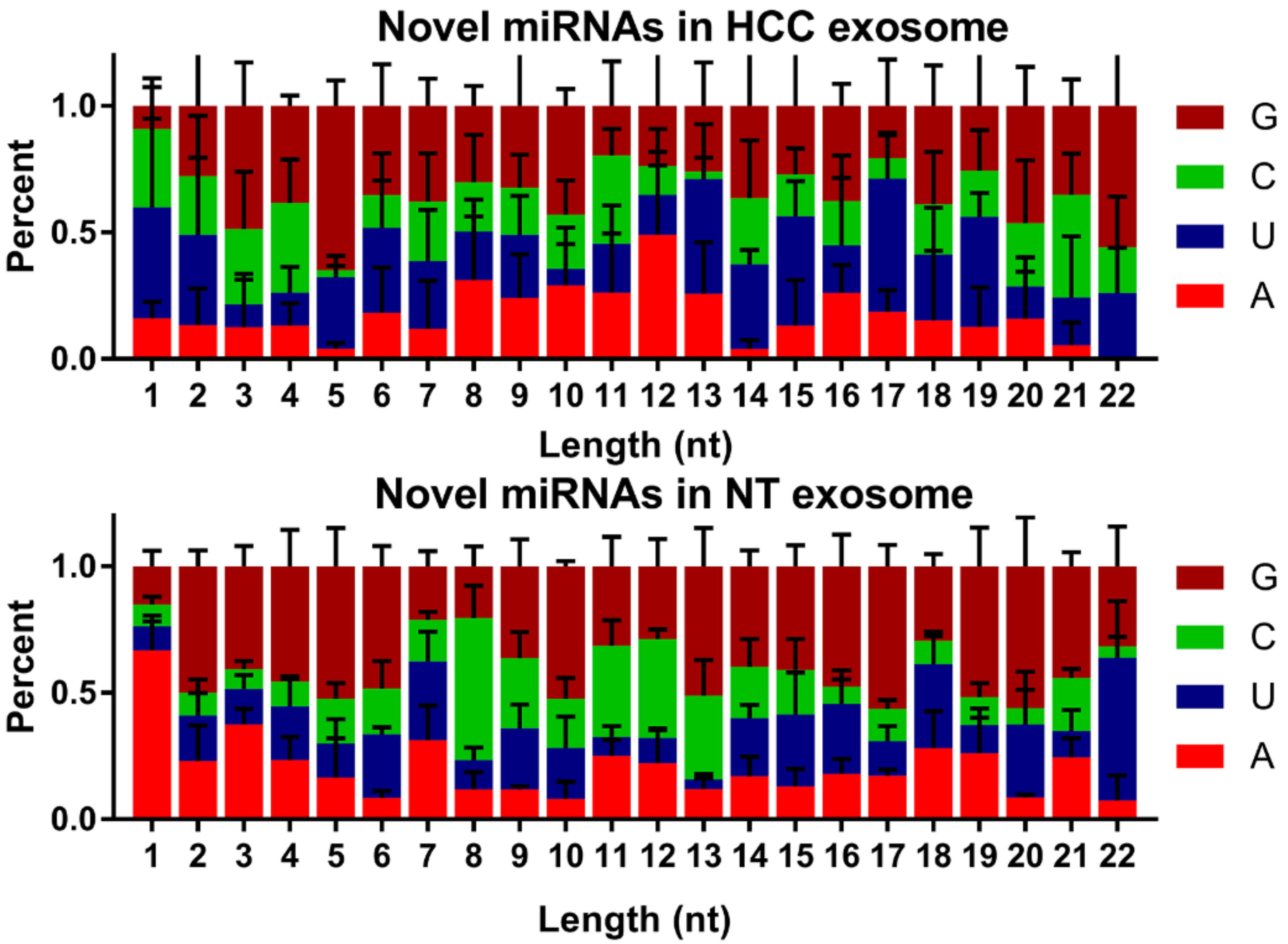

Figure S10: Base bias of novel miRNAs from serum exosomes in 4 HCC patients and 4 non-tumor donors.

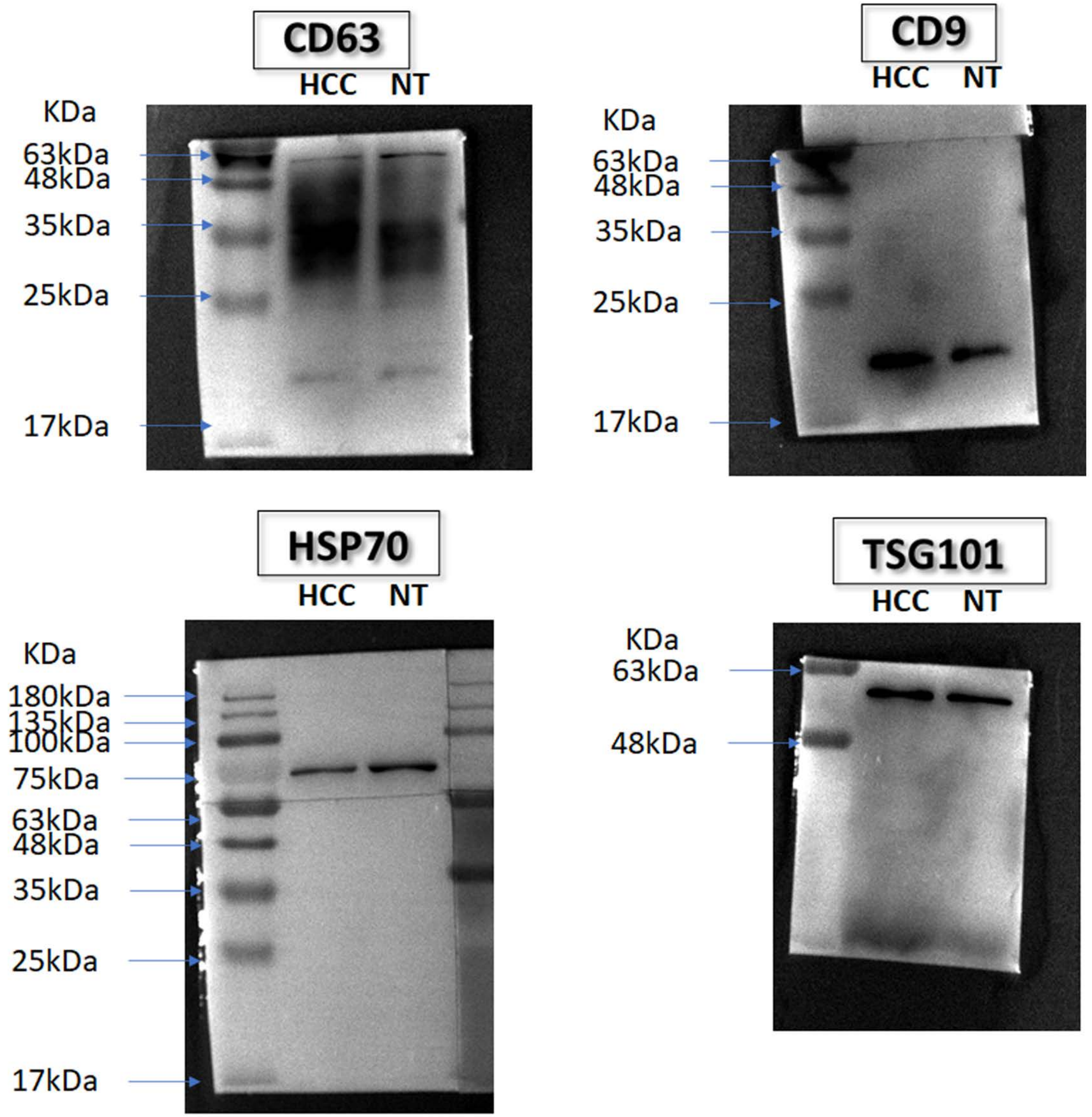

Figure S11 The Original whole Western blot

Table.S1: Patient characteristics

Characteristic	
Age	59.60 ± 8.56
≤ 65	35 (28.2%)
> 65	89 (71.8%)
Gender, n (%)	
Male	90 (72.6%)
Female	34 (27.4%)
HBV infection, n (%)	
Negative	30 (24.2%)
Positive	94 (75.8%)
AFP, n (%)	
≤ 100 ng/ml	78 (62.9%)
> 100 ng/ml	46 (37.1%)
Tumor size, n (%)	6.61 ± 2.53
≤ 5 cm	41 (33.1%)
> 5 cm	83 (66.9%)
T-Stage, n (%)	
T1	59 (47.6%)
T2	10 (8.1%)
T3	2 (1.6%)
T4	53 (42.7%)
Low (T1+T2)	68 (54.4%)
High (T3+T4)	57 (45.6%)
AJCC-Stage, n (%)	
Stage1	57 (46.0%)
Stage2	8 (6.5%)
Stage3	48 (38.7%)
Stage4	11 (8.9%)
Low (Stage 1+2)	65 (52.4%)
High (Stage 1+2)	59 (47.6%)
Tumor number, n (%)	
Isolated	109 (87.9%)
multiple	13 (10.5%)
Macrovascular invasion, n (%)	
Negative	69 (55.6%)
Positive	55 (44.4%)
HCV infection, n (%)	
Negative	124 (100.0%)
Positive	0 (0.0%)
Liver cirrhosis, n (%)	
Negative	84 (67.7%)
Positive	40 (32.3%)
Hypertension, n (%)	

Negative	81 (65.3%)
Positive	43 (34.7%)
Diabetes mellitus, n (%)	
Negative	78 (62.9%)
Positive	46 (37.1%)
Ascites, n (%)	
Negative	78 (62.9%)
Positive	46 (37.1%)
Child-pugh	
A	118 (95.2%)
B	6 (4.8%)
C	0 (0%)
Total bilirubin (μmol/L)	15.42 ± 18.83
diabetes mellitus (mmol/L)	5.32 ± 1.17
Gamma glutamyl transferase (U/L)	95.14 ± 108.86
Albumin (g/L)	38.06 ± 4.586
Prothrombin time (s)	11.79 ± 0.94
International normalized ratio	0.99 ± 0.09
Alanine aminotransferase (U/L)	39.19 ± 32.09
Aspartate aminotransferase (U/L)	45.30 ± 50.10

Table.S2:

The characteristics of **4 randomly selected patients** for sRNA-sequencing.

Characteristics	Patient 1	Patient 2	Patient 3	Patient 4
Age (y)	65	58	71	54
Gender	Male	Male	Male	Female
HBV DNA	Positive	Positive	Negative	Positive
AFP (ng/ml)	2790.1	3.9	2.6	4.0
Cirrhosis	Negative	Positive	Negative	Negative
TBil (μmol/L)	25.3	17.2	24.4	16.5
Albumin (g/L)	49.2	39.7	36.7	45.4
Prothrombin time (s)	11.7	12.4	11.8	11.7
Ascites	Negative	Negative	Negative	Negative
Hepatic encephalopathy	Negative	Negative	Negative	Negative
Tumor size (cm)	9*7*7	3.6*3.2*3.2	5.3*4.2*3.2	3*2.5
Tumor number	Isolated	Isolated	Isolated	Multiple
Node metastasis	Negative	Negative	Negative	Positive
Distant metastasis	Negative	Negative	Negative	Negative
Macrovascular invasion	Positive	Negative	Positive	Negative
Tumor location	Left lobe	Right lobe	Right lobe	Right lobe

The characteristics of **4 randomly selected non-tumor donors** for sRNA-sequencing.

Characteristics	donor 1	donor 2	donor 3	donor 4
Age (y)	38	66	79	69
Gender	Female	Female	Male	Male
HBV DNA	Negative	Negative	Negative	Negative
AFP (ng/ml)	2.3	3.5	3.1	2.9
Cirrhosis	Negative	Negative	Negative	Negative
TBil (μmol/L)	3.6	6.8	11.6	20.3
Albumin (g/L)	43.9	46.3	45.2	48.4
Prothrombin time (s)	12.1	11.9	12.5	11.8
Ascites	Negative	Negative	Negative	Negative
Hepatic encephalopathy	Negative	Negative	Negative	Negative
Tumor size (cm)	Not applicable	Not applicable	Not applicable	Not applicable
Tumor number	Not applicable	Not applicable	Not applicable	Not applicable
Node metastasis	Not applicable	Not applicable	Not applicable	Not applicable
Distant metastasis	Not applicable	Not applicable	Not applicable	Not applicable
Macrovascular invasion	Not applicable	Not applicable	Not applicable	Not applicable
Tumor location	Not applicable	Not applicable	Not applicable	Not applicable

Table.S3. The sequences of primers for miRNA amplification.

Name	Sequence
miR-122-5p RT loop primer	GTCGTATCCAGTGCAGGGTCCGAGGTATTCGCACTGGATA CGACCAAACA
miR-122-5p Foward primer	GCAGATGTGACCTTCTCG
let-7d-5p RT loop primer	GTCGTATCCAGTGCAGGGTCCGAGGTATTCGCACTGGATA CGACAACTAT
let-7d-5p Foward primer	GCCGGGAGAGGTAGTAGGTTGC
miR-425-5p RT loop primer	GTCGTATCCAGTGCAGGGTCCGAGGTATTCGCACTGGATA CGACTCAACG
miR-425-5p Foward primer	GCGAATGACACGATCACTCC
Reverse primer for miRNA	CAGTGCAGGGTCCGAGGTAT

Table.S4. Antibodies for Western blot.

Name	Catalog	Molecular weight (kDa)
anti-TSG101	A5789, Abclonal	44
anti-CD9	A19027, Abclonal	25
anti-HSP70	A1507, Abclonal	70
anti-CD63	A5271, Abclonal	25-50
anti-Rabbit IgG HRP-link	7074, CST	
anti-Mouse IgG HRP-link	7076, CST	

Table.S5:

The diagnostic performance characteristics from 3 miRNAs **in training set**.

Characteristics	miR-122-5p	miR-425-5p	let-7d-5p	3-miRNA
Sensitivity	0.84 (0.75-0.92)	0.83 (0.75-0.92)	0.58 (0.47-0.69)	0.87 (0.79-0.95)
Specificity	0.85 (0.76-0.93)	0.45 (0.34-0.56)	0.85 (0.77-0.93)	0.85 (0.77-0.93)
PPR	0.94 (0.88-0.99)	0.81 (0.72-0.90)	0.91 (0.85-0.98)	0.94 (0.89-0.99)
NPR	0.65 (0.54-0.76)	0.50 (0.39-0.61)	0.43 (0.31-0.53)	0.71 (0.61-0.81)
PLR	5.58 (0-11.34)	1.52 (1.34-1.70)	3.88 (1.35-6.41)	5.82 (0-12.1)
NLR	0.19 (0.10-0.28)	0.36 (0.25-0.47)	0.49 (0.38-0.61)	0.14 (0.07-0.23)
Miss rate	0.16 (0.08-0.25)	0.16 (0.08-0.25)	0.42 (0.30-0.53)	0.13 (0.05-0.20)
MR	0.15 (0.07-0.23)	0.55 (0.44-0.66)	0.15 (0.07-0.23)	0.15 (0.07-0.23)

PPR: Positive predictive rate; NPR: Negative predictive rate; PLR: Positive likelihood radio; NLR: Negative likelihood radio; MR: Misdiagnosis rate

The diagnostic performance characteristics from 3 miRNAs **in validation set**.

Characteristics	miR-122-5p	miR-425-5p	let-7d-5p	3-miRNA
Sensitivity	0.87 (0.80-0.93)	0.74 (0.66-0.83)	0.41 (0.32-0.51)	0.89 (0.82-0.95)
Specificity	0.88 (0.82-0.95)	0.54 (0.44-0.64)	0.92 (0.86-0.97)	0.92 (0.87-0.98)
PPR	0.95 (0.91-0.99)	0.81 (0.73-0.89)	0.93 (0.89-0.98)	0.97 (0.93-1.00)
NPR	0.72 (0.63-0.81)	0.44 (0.34-0.54)	0.36 (0.27-0.47)	0.75 (0.66-0.84)
PLR	7.55 (0-17.45)	1.61 (1.41-1.80)	5.38 (0.66-10.11)	11.51 (0-35.7)
NLR	0.14 (0.07-0.21)	0.48 (0.38-0.58)	0.63 (0.54-0.73)	0.12 (0.05-0.19)
Miss rate	0.13 (0.06-0.19)	0.26 (0.17-0.34)	0.59 (0.49-0.68)	0.11 (0.05-0.18)
MR	0.12 (0.05-0.18)	0.46 (0.36-0.56)	0.08 (0.02-0.14)	0.07 (0.02-0.13)

PPR: Positive predictive rate; NPR: Negative predictive rate; PLR: Positive likelihood radio; NLR: Negative likelihood radio; MR: Misdiagnosis rate

Table.S6 The correlation between three exo-miRNAs and clinicopathological characteristics of HCC.

Charactristics	exo-miR-122-5p (n)			exo-miR-425-5p (n)			exo-let-7d-5p (n)		
	Low	High	p	low	High	p	low	High	p
Age(y)									
< 65	24	25	0.633	22	25	0.396	24	25	0.398
≥ 65	10	8		10	7		10	8	
Gender									
Female	7	5	0.562	6	5	0.740	7	5	0.523
Male	27	28		26	27		26	28	
AFP (ng/ml)									
≥ 400	25	22	0.539	23	22	0.784	21	26	0.174
< 400	9	11		9	10		12	7	
HBV DNA									
Negative	8	9	0.725	8	7	.0768	11	5	0.085
Positive	26	24		24	25		22	28	
Tumor size (cm)									
≥ 5	12	10	0.664	11	11	1.000	9	13	0.296
< 5	22	23		21	21		24	20	
T stage									
Low	17	19	0.534	16	18	0.616	13	22	0.026
High	17	14		16	14		20	11	
AJCC stage									
Low	16	18	0.540	14	18	0.317	12	21	0.027
High	18	15		18	14		21	12	
Tumor location									
Left	8	7	0.821	7	8	0.767	5	10	0.140
High	25	25		24	23		27	22	
Tumor number									
Isolated	30	29	1.000	29	27	0.247	29	29	1.000
Multiple	3	4		2	5		4	3	
Macrovascular invasion									
Negative	17	19	0.534	16	18	0.616	14	21	0.084
Positive	17	14		16	14		19	12	
